# Late‐Life depression exacerbates cognitive decline and Tau pathology in an Alzheimer's disease model

**DOI:** 10.1002/alz70855_106524

**Published:** 2025-12-24

**Authors:** Laura Vegas‐Gomez, Jesus Garcia‐Martin, Maria Angeles Arredondo‐Alcala, Antonia Gutierrez, Ines Moreno‐Gonzalez

**Affiliations:** ^1^ Dept. Cell Biology, Faculty of Sciences, University of Malaga. IBIMA., Malaga, Spain; ^2^ Centro de Investigación Biomédica en Red (CIBER), Madrid, Spain; ^3^ The University of Texas Health Science Center at Houston, Houston, TX, USA

## Abstract

**Background:**

Recent studies suggest that depression may be a crucial risk factor for the development of cognitive impairment and Alzheimer's disease (AD). In fact, there is a strong association between late‐life depression and AD. The age of AD onset has been shown to be accelerated in patients with mild cognitive impairment (MCI) with a history of depression, and women appear to be particularly more vulnerable to this condition. In addition, individuals with MCI who present depressive symptoms have an elevated burden of amyloid‐beta, the main toxic protein associated with AD pathology, and a higher risk of developing AD compared to non‐depressed MCI patients. Although it has been described that some transgenic models of AD can develop signs similar to depression in advanced stages, the induction of AD pathology due to a depressive process has not been studied under experimental conditions to emulate late‐life depression as a risk factor for dementia

**Method:**

The objective of this study is to determine, by inducing unpredictable mild chronic stress (CUMS) in tau transgenic P301S mice, whether depression is a cause, rather than a consequence, of AD development.

**Result:**

The results of our study indicate that the induction of CUMS in transgenic animals induces phenotypic changes related to a depressive state. Behavioral and histological studies suggest that depression‐like induction can worsen AD pathology.

**Conclusion:**

The findings generated in this project could provide evidence of depression as a risk factor for AD, its mechanisms of action, use as early biomarkers, as well as the discovery of new therapies for AD

